# The Danube Delta: A natural laboratory for trans-disciplinary education, research, innovation and development

**Published:** 2014

**Authors:** Sorel Eliot

**Affiliations:** * The George Washington University, Washington, DC, USA

**Abstract**

**The Danube Delta**

• A European and global ecological treasure

• A rich, diverse flora & fauna

• 98% of the European aquatic fauna

• 3400 animal species

• 160 fish species

• 300 bird species – Arctic, Siberian, Mongolian, Chinese, Mediterranean, European

• Abundant and diverse natural resources

• 1150 plant species

• Over 4178 square Km Delta

• A Danube Delta Biosphere of 5800 square Km 

• Significant cultural diversity

• Source of energy

• Corridors of energy transport

• The Danube from the Black Forest to Caraorman Forest

• Romania & the Danube Delta

• Bridge between EU, the Black Sea region

• Regions with diversity of cultures and traditions

• Rich in natural & human resources

• Aspiring for higher education, development

• Ties with the EU and the Euro-Atlantic communities

**The Danube Delta Project**

• The *Danube Delta Institute of Advanced Trans-Disciplinary Studies (DDIATS)*

• The *Danube Delta International Summer School of ATS (DDISSATS)*

• To stimulate knowledge generation, translation, transmission

• Application, via education, research, innovation, development

• Human capital development

• To close the development gap between the region and the world

• Open up new opportunities for collaboration among

• Students, scholars, professionals, business, policy makers, 

• Of the region as well as with those from EU and USA

• Across disciplines & continents

• Focusing on history, cultures, & traditions

• Ecology

• Energy 

• Health systems’ strengthening

• Technology, green growth, and sustainable development

• Identify and secure educational, human, financial resources

• For bilateral & multilateral aspects of the projects

• Specify all resources necessary for implementation

• Involve public, private, institutions, governments, foundations, & benefactors for financing the projects

• Produce a projects’ hierarchy & implementation timeline

• Agree on a leadership workgroup

**The Danube Delta Project**

Anticipated Outcomes

• Knowledge generation, innovation, human capital development

• Energy, ecology, green growth, sustainable development

• Science, technology, innovation, & health systems’ strengthening Measuring societies’ progress in tandem with OECD

**Keywords:** Danube Delta, trans-disciplinary education, research, innovation

**The Danube Delta & Environs**

• Limited knowledge of the Delta, Black Sea regions 

• In the Euro-Atlantic educational, research and policy communities

• The Danube Delta trans-disciplinary project

• Involving universities, the business community & NGOs

**The Danube Delta Project**

• The *Danube Delta Institute of Advanced Trans-Disciplinary Studies (DDIATS)*

• The *Danube Delta International Summer School of ATS (DDISSATS)*

• To stimulate knowledge generation, translation, transmission

• Application, via education, research, innovation, development

• Human capital development

• To close the development gap between the region and the world

• Open up new opportunities for collaboration among

• Students, scholars, professionals, business, policy makers, 

• Of the region as well as with those from EU and USA

• Across disciplines & continents

• Ecology

• Energy 

• Health systems’ strengthening

• Technology, green growth, and sustainable development

• Identify and secure educational, human, financial resources

• For bilateral & multilateral aspects of the projects

• Specify all resources necessary for implementation

• Involve public, private, institutions, governments, foundations, & benefactors for financing the projects

• Produce a projects’ hierarchy & implementation timeline

• Agree on a leadership workgroup

**The Danube Delta Project**

Anticipated Outcomes

• Knowledge generation, innovation, human capital development

• Energy, ecology, green growth, sustainable development

• Science, technology, innovation, & health systems’ strengthening Measuring societies’ progress in tandem with OECD

**Energy, Ecology, & Health**

A Public/Private/NGOs Partnership:

• Governments-ministries of health, education, defense

• Academic centers, universities, professional organizations

• OECD, EU CDC, US CDC, USAID, WB,WHO, NATO

• European Observatory on Health Systems

• business community, oil, gas, alternative energy industries

• Foundations and NGOs

• The media

• Ecology – water, air, and soil safety

• Energy & Ecology

• deep sea gas & oil exploration, preventing disasters

• fossil fuel v. wind, solar, geo, other

• Health

• Communicable diseases (CD) Avian flue prevention project

• Non-communicable diseases (NCDs) & ICT projects

• Health systems’ management & strengthening

• Enhance educational, employment & scholarly opportunities for

• Young people, scholars, development communities of the region

• Enhanced collaboration with counterparts in the EU & USA

• Close the knowledge, understanding, & action gaps between

• The region, the Euro-Atlantic community

• Deliver innovative, education, research, development projects 

• The free flow of people, ideas, goods, & services

**Innovative Educational Laboratory**

• 21st Century educational transformations

• A dynamic process of teacher & student learning together

• In a relationship of trust

• Teaching/Learning values & criteria rather than figures & facts

• Think, decide, make choices rather than memorize

• Generating trust through promoting students’ own initiatives

• Stimulating their creative impulses, ingenuity, imagination

• Embracing information communication technology (ICT)

• Discern fact from fiction using empirical & scientific evidence

• Technology is no substitute for the individual’s ability to reason

• Intellectual & ethical tools complimenting each other

• Value system-based decisions

• Inspiring, leadership, commitment generating, not authoritarian

• An education that puts some heart into thought

• Preparing students not for a job, but for any job 

• How to obtain knowledge about any issue

• Understanding & applicability of interdependent variables 

• Systemic, systematic & integrative thinking & acting

• Mindful of the ecology, energy, health, development equilibrium

• Profit motives and societal responsibilities

**The Danube Delta Project’s Evaluation**

DDIATS & DDISSATS

• Transparent, qualitative & quantitative

• Educational, research, services, development projects

• Annual public reports, recommendations, & follow-up actions

• Each projects’ annual report in the English language, and in

• The languages of all partners

• Posted on a timely & regular basis on all partners’ websites

• Open to feedback from all involved and affected by the projects
